# Progress and challenges in CRISPR/Cas applications in microalgae

**DOI:** 10.71150/jm.2501028

**Published:** 2025-03-28

**Authors:** Quynh-Giao Tran, Trang Thi Le, Dong-Yun Choi, Dae-Hyun Cho, Jin-Ho Yun, Hong Il Choi, Hee-Sik Kim, Yong Jae Lee

**Affiliations:** 1 Cell Factory Research Center, Korea Research Institute of Bioscience and Biotechnology (KRIBB), Daejeon 34141, Republic of Korea; 2 Department of Environmental Biotechnology, KRIBB School of Biotechnology, University of Science and Technology (UST), Daejeon 34113, Republic of Korea

**Keywords:** CRISPR/Cas, microalgae, synthetic biology, genome editing, off-target effect, transformation efficiency

## Abstract

Clustered Regularly Interspaced Short Palindromic Repeats (CRISPR) technologies have emerged as powerful tools for precise genome editing, leading to a revolution in genetic research and biotechnology across diverse organisms including microalgae. Since the 1950s, microalgal production has evolved from initial cultivation under controlled conditions to advanced metabolic engineering to meet industrial demands. However, effective genetic modification in microalgae has faced significant challenges, including issues with transformation efficiency, limited target selection, and genetic differences between species, as interspecies genetic variation limits the use of genetic tools from one species to another. This review summarized recent advancements in CRISPR systems applied to microalgae, with a focus on improving gene editing precision and efficiency, while addressing organism-specific challenges. We also discuss notable successes in utilizing the class 2 CRISPR-associated (Cas) proteins, including Cas9 and Cas12a, as well as emerging CRISPR-based approaches tailored to overcome microalgal cellular barriers. Additionally, we propose future perspectives for utilizing CRISPR/Cas strategies in microalgal biotechnology.

## Introduction

Microalgae are photosynthetic microorganisms that play a crucial role in mitigating climate change by effectively capturing carbon dioxide (Onyeaka et al., 2021). To produce one kilogram of biomass, up to two kilograms of CO_2_ are consumed when microalgae are grown under autotrophic conditions using sunlight as an energy source (Fernández et al., 2021). In addition, many microalgal species can thrive in heterotrophic conditions, which is favorable for industrial production due to easy control and stable output. Studies have shown that microalgae can achieve growth rates ranging from one to three doublings per day, depending on the species, under heterotrophic conditions (Bumbak et al., 2011). For instance, fast-growing species such as *Chlorella*, *Crypthecodinium*, *Nitzia*, and *Prototheca* can exhibit specific growth rates exceeding 0.09 h^-1^, allowing them to double in less than 7.7 h. In contrast, other species such as *Galdieria*, *Haematococcus*, *Nannochloropsis*, and *Schizochytrium* typically grow at approximately half that rate, doubling every 10 to 100 h (Bumbak et al., 2011). Compared to higher plants, microalgae can complete an entire growth cycle in just a few days. Yet, common industrial organisms such as yeast and bacteria often achieve growth rates surpassing 15 doublings per day under optimal conditions. For example, common *Escherichia coli* strains such as K12 and BL21 have specific growth rates of 0.63–0.68 h^-1^, whereas the model yeast strain *Saccharomyces cerevisiae* S288C can achieve a specific growth rate of 0.42 h^-1^ (Long et al., 2024). From an industrial point of view, the development of fast-growing algal strains is essential to facilitate their large-scale applications.

In addition to their ecological contributions, microalgae currently contribute to the global economy, with a market size of up to 100 kilotons per year and biomass prices ranging from €5 to €500 per kilogram, mainly in human-related applications such as food, animal feed, pharmaceuticals, and cosmetics (Fernández et al., 2021). Notable microalgal species, such as *Spirulina* and *Chlorella*, have been widely recognized and commercialized as dietary supplements in powder, tablet, and capsule forms (Ye et al., 2018). Their nutritional profile, characterized by high protein content, essential fatty acids such as omega-3, and antioxidants such as carotenes and chlorophylls, makes them indispensable in the field of health and wellness (Abreu et al., 2023). Furthermore, microalgae hold promise in many emerging industrial applications. For instance, they have been recognized as a third-generation bioenergy source with a lower environmental impact than traditional fossil fuels (Chowdhury & Loganathan, 2019). A landmark moment occurred in 2009 when algae-derived jet fuel successfully powered a twin-engine commercial jet without engine modifications (Adeniyi et al., 2018). Despite this progress, the future commercialization of microalgal biomass for energy production remains unpredictable. This uncertainty arises mainly from the high costs associated with cultivation and processing, alongside limited biomass productivity (Qin et al., 2023). Similarly, although microalgae-based bioplastic production offers environmentally friendly and biodegradable alternatives to conventional fossil-based plastics, it is still in the research and development stage and is far from being commercialized. In addition to biomaterials, microalgae can play an important role in agriculture by producing biofertilizers and biostimulants (Fernández et al., 2021). These biofertilizers enhance sustainable farming practices by reducing the need for chemical fertilizers while providing beneficial phytohormones that promote crop growth (Zhang et al., 2024). A key factor in realizing the full potential of microalgae in these fields is the advancement of genetic engineering tools. Although considerable progress has been made in the biotechnological manipulation of microalgae, current methodologies still lag behind those applied to other microorganisms. Genetic modifications offer the potential to enhance growth rates, lipid production, CO_2_ sequestration, and synthesis of valuable bioproducts. By targeting specific genes involved in lipid and carbohydrate metabolism, researchers can increase the productivity and growth rates of microalgae, making them a more competitive alternative in the industry.

Since the 1960s, random mutagenesis has been used as a simple technique to generate mutants in microalgae, focusing on observable phenotypes rather than targeting specific gene modifications (Trovão et al., 2022). Researchers have successfully created a colorless *Chlorella* mutant by exposing it to radioactive isotopes, which lacked both carotenoids and chlorophyll but exhibited increased respiratory activity when illuminated with blue light (Schmid & Schwarze, 1969). Over the years, random mutagenesis has received increasing attention for its application in various microalgae, including *Nannochloropsis* (Ma et al., 2013), *Chlorella* sp. (Manandhar-Shrestha & Hildebrand, 2013), *Tetraselmis* sp. (Dinesh Kumar et al., 2018), and *Phaeodactylum tricornutum* (Macdonald Miller et al., 2023). This approach has proven effective in enhancing biomass production, increasing the yields of target compounds, and improving strain tolerance to diverse environmental conditions, thereby contributing to the development of high-yielding microalgal strains. Similarly, adaptive laboratory evolution (ALE) is a promising approach that employs random mutation and natural selection to improve the performance and beneficial traits of microalgae (Zhang et al., 2021). However, both random mutagenesis and ALE are labor-intensive and time-consuming processes, often requiring multiple generations to achieve the desired phenotype (Trovão et al., 2022).

**Fig. 1.**
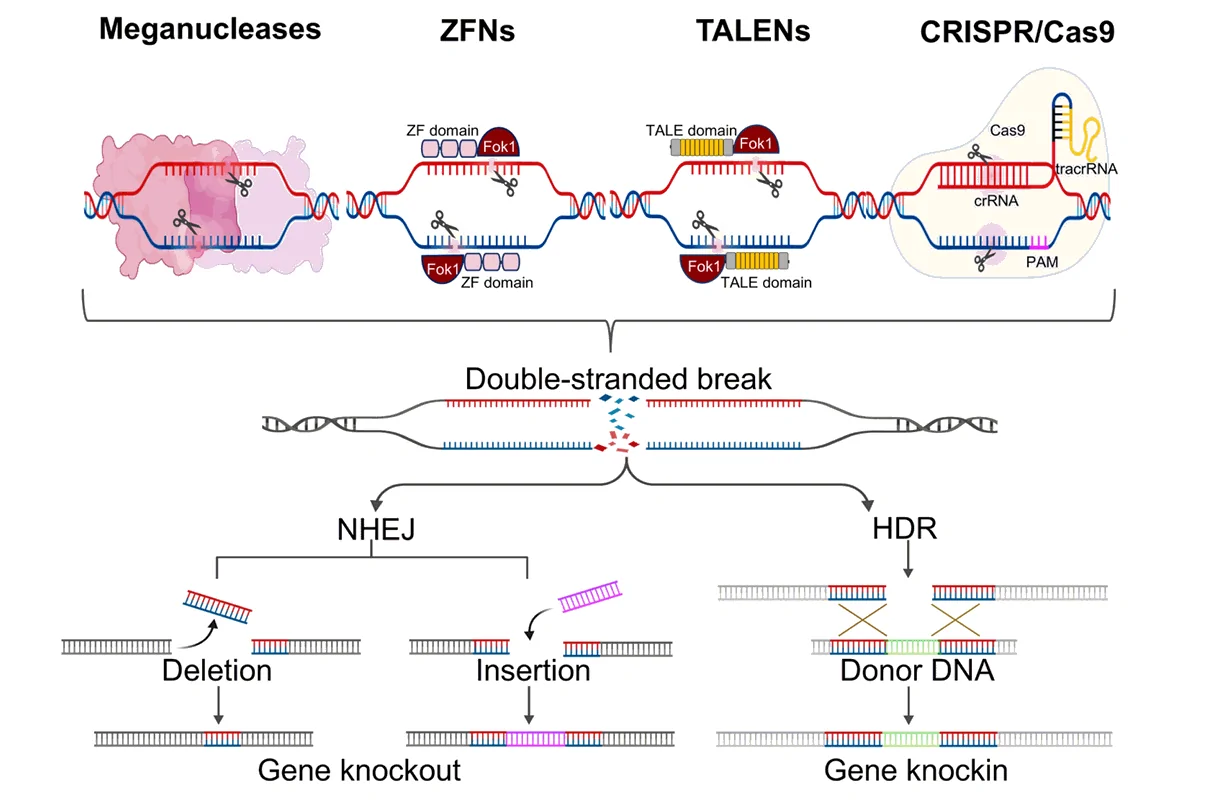
Genome editing strategies and DNA repair mechanisms in microalgae. Meganucleases, ZFNs, TALENs and CRISPR/Cas9 enable precise and efficient genome modification by inducing double-strand breaks, which can be repaired by non-homologous end joining (NHEJ) or homology-directed repair (HDR). NHEJ-mediated repair typically results in variable-length insertions or deletions. HDR can result in point mutations or gene replacements in the presence of donor DNA. ZFNs, zinc-finger nucleases; TALENs, transcription activator-like effector nucleases; CRISPR/Cas9, clustered regularly interspaced short palindromic repeats and CRISPR-associated protein 9; crRNA, CRISPR RNA; tracrRNA, trans-activating CRISPR RNA; NHEJ, non-homologous end joining; HDR, homology-directed repair.

Recent advancements in synthetic biology have demonstrated great promise in facilitating precise genomic modification in microalgae (Lu et al., 2021). Over the past decade, genome editing techniques have progressed rapidly, enabling precise editing of nearly any desired DNA sequence. Techniques such as site-directed nucleases, including meganucleases, Zinc-Finger Nucleases (ZFNs), Transcription Activator-Like Nucleases (TALENs), and Clustered Regularly Interspaced Short Palindromic Repeats (CRISPR)/CRISPR-associated (Cas) endonucleases induce double-stranded breaks in DNA, stimulating the cell's inherent DNA repair mechanisms (Gaj et al., 2013; Silva et al., 2011). This process can lead to gene knockouts or facilitate the insertion of transgenes when combined with donor DNA (Fig. 1). Initial attempts have engaged meganucleases, ZFNs, and TALENs for gene editing in various microalgal species, including *P. tricornutum* (Daboussi et al., 2014) and *Chlamydomonas reinhardtii* (Sizova et al., 2013). However, the necessity to design specific proteins to recognize target genes adds considerable complexity, resulting in time constraints that hinder the practicality of these methods for microalgal genome engineering.

In recent years, CRISPR/Cas technologies have gained prominence in microalgal genome editing, primarily due to their versatility and high specificity. The CRISPR/Cas9 system, originally characterized as a bacterial adaptive immune mechanism against invading plasmids and viral DNAs, operates by cleaving target DNA molecules guided by small RNA sequences (Adli, 2018). This system comprises three principal components: the Cas9 nuclease protein, the complementary base-pairing CRISPR RNA (crRNA) containing the target sequence (protospacer), and the transactivating CRISPR RNA (tracrRNA) that forms a secondary scaffold structure recognized by Cas9. When a small linker sequence is inserted, the crRNA and tracrRNA can be expressed as a single guide RNA (sgRNA), which binds to the Cas9 nuclease to form a Cas9/sgRNA ribonucleoprotein (RNP) complex. Since its introduction for genome editing, the CRISPR/Cas9 system has successfully knockout genes in various model organisms (Li et al., 2023). Nonetheless, employing this system for genome editing in microalgae encounters significant challenges. A primary obstacle is the effective delivery of necessary components, specifically the Cas9 protein and guide RNA (gRNA), into microalgal cells. The rigid cell walls of microalgae vary in composition and thickness among species, posing substantial barriers to the efficient transformation and uptake of exogenous materials (Ortiz-Matamoros et al., 2017). Moreover, interspecies genetic variability presents another challenge, as genetic tools developed for one species may not apply effectively to another. Fortunately, the declining costs of genome sequencing technologies now enable in-depth characterization of a broader array of microalgal strains, facilitating the tailored adaptation of genetic tools to align with the specific codes of targeted species.

This review aims to summarize current insights into the factors influencing the success of CRISPR/Cas technology in microalgal research. Specifically, we will focus on the following aspects: (i) the CRISPR components used in microalgae compared to other organisms, (ii) the challenges and potential solutions for effective gene editing, and (iii) the applications of CRISPR/Cas technologies in the realm of microalgal synthetic biology.

## Milestones in the Application of CRISPR/Cas System in Microalgae

**Fig. 2.**
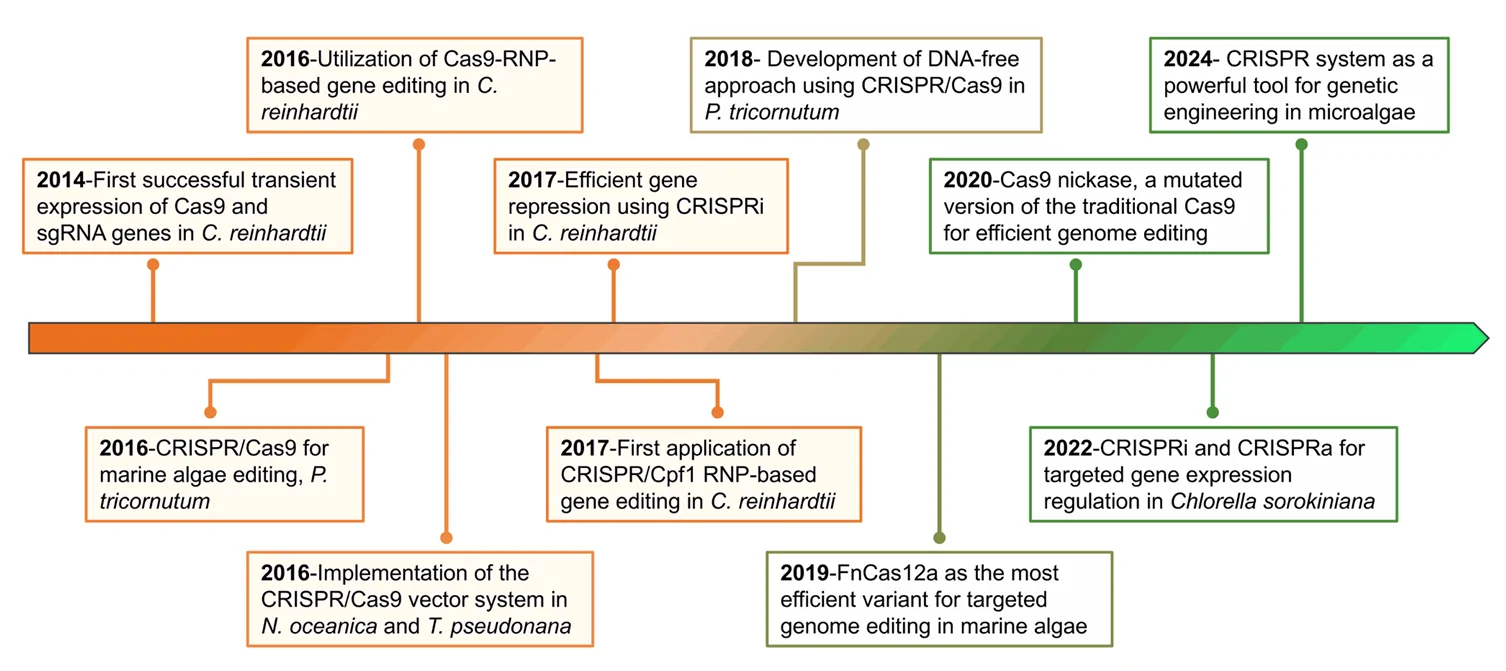
Milestones in the application of CRISPR/Cas system in microalgae.

The pioneering application of the CRISPR/Cas9 system in microalgae was first reported by Jiang et al. (2014), representing a case study of precise gene editing in *C. reinhardtii*. By expressing a vector containing both the Cas9 protein and sgRNA, the researchers successfully targeted and disrupted the *FKB12* gene, which is involved in rapamycin sensitivity. The successful knockout of a specific gene in *C. reinhardtii* demonstrated the feasibility of applying CRISPR/Cas9 to microalgae and encouraged further research in this area (Fig. 2). However, targeted modification of the *FKB12* gene produced only a single rapamycin-resistant colony with the desired genetic modification from extensive efforts involving more than 10^9^ cells in 16 independent transformation experiments (Jiang et al., 2014). This outcome highlighted the challenges in utilizing the CRISPR/Cas9 system in microalgae, particularly the potential cytotoxic effects resulting from the constitutive expression of the CRISPR components. In their subsequent study, Jiang and Weeks (2017)  worked on improving the performance of the plasmid-based Cas9 system in *C. reinhardtii*. They introduced a hybrid expression construct that combined a Cas9 gene with an artificial intron and an inserted sgRNA gene. This innovation yielded 13 colonies from four transformations, marking an efficiency improvement to about 1 colony per 3 × 10^7^ initial cells. However, Cas9 toxicity still appeared to be prevalent.

The landscape for CRISPR/Cas applications in microalgae continued to evolve over the following years. Notably, researchers have successfully employed a plasmid vector containing a diatom-specific codon-optimized Cas9 to knock out the CpSRP54 gene in *P. tricornutum* (Nymark et al., 2016). This study also marked the first successful application of CRISPR/Cas in marine diatoms. Additionally, they introduced a high-resolution PCR-based method to screen for mutations, achieving a mutation frequency of 31% across 26 transformants. Just a few months later, Baek et al. (2016) and Shin et al. (2016) reported the use of Cas9 RNP complexes in *C. reinhardtii*, addressing previous challenges of cytotoxicity associated with vector-driven expression. By delivering preassembled Cas9 proteins and sgRNAs directly into cells, they efficiently induced mutations at different loci with varying mutation frequencies, with non-homologous end joining (NHEJ)-mediated knockin events identified at Cas9 cut sites (Shin et al., 2016). Baek et al. (2016) achieved mutations between 0.46% and 1.1% when attempting single and double-gene knockouts. These methods not only improve mutagenesis efficiency but also reduce the time required to generate conventional knockout mutants.

The successful implementation of the CRISPR/Cas9 vector system was further reported across various microalgal species, including *Nannochloropsis oceanica*, a model marine microalga for biofuel production (Wang et al., 2016), and *Thalassiosira pseudonana*, a model centric diatom recognized for its ecological and biotechnological significance (Hopes et al., 2016). In the case of *N. oceanica*, the researchers achieved a success rate of approximately 1/1000 to 1/100 in generating knockout mutants of the nitrate reductase (*NR*) gene, representing an improvement in efficiency several orders of magnitude greater than that observed in *C. reinhardtii*. They also introduced a novel screening method involving restriction enzyme digestion, nested PCR, and deep sequencing to reliably identify genome-edited mutants (Wang et al., 2016). For *T. pseudonana*, only the promoters and target sequences were species-specific, while *Streptococcus pyogenes* Cas9 (*Sp*Cas9), which has human codon bias and carries the SV40 nuclear localization signal, was used. The researchers successfully utilized multiple sgRNAs targeting a single gene, thus achieving a high rate of bi-allelic mutants with precise deletions (Hopes et al., 2016).

In 2017, the focus shifted to applying preassembled CRISPR/Cpf1 RNPs (also known as Cas12a) for gene editing. Ferenczi et al. (2017) reported the use of Cpf1 RNP, together with single-stranded DNA repair templates, to achieve precise and targeted DNA replacement with up to 10% efficiency in a cell wall-deficient *C. reinhardtii* strain, where it generated NHEJ-mediated indels at a frequency of 0.02%. The following year, Naduthodi et al. (2019) tested different Cas12a variants, achieving a remarkable 93% efficiency for generating homology-directed repair (HDR)-based targeted mutations using *Fn*Cas12a (from *Francisella novicida*) in *N. oceanica* IMET1. They demonstrated that co-delivering Cas RNPs with a double-stranded DNA (dsDNA) repair template significantly enhanced homologous recombination (HR) in this marine alga.

**Fig. 3.**
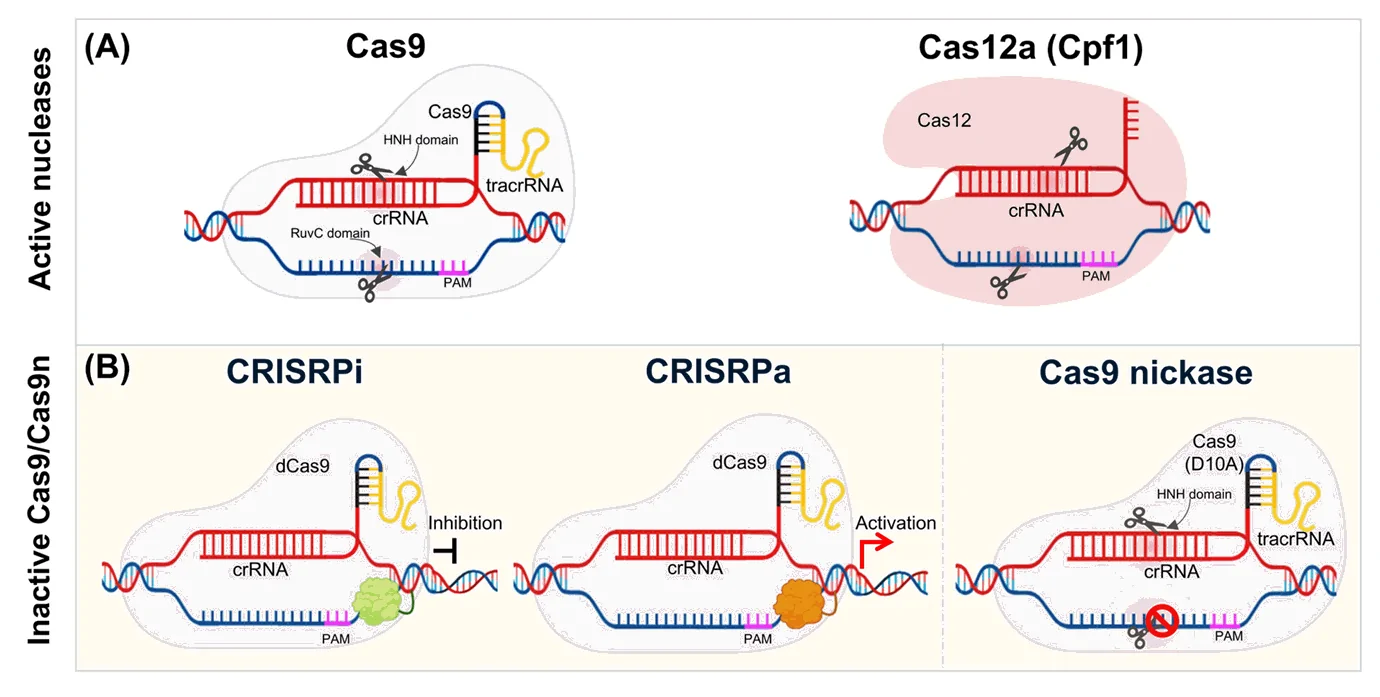
Schematic diagram of various Cas nucleases used in microalgae. (A) Cas9 cleaves target DNA at two nuclease domains (HNH and RuvC), facilitated by the binding of a sgRNA, which is a combination of crRNA and tracrRNA, to the target DNA strand near the protospacer adjacent motif (PAM) site and produce blunt end of double strand break (DBS). Cas12a cleaves target DNA at specific sites guided by crRNA nearby PAM site to produce sticky end of DSB. (B) CRISPRi uses dCas9 protein fused to a transcriptional repressor (green) to inhibit gene expression while CRISPRa uses dCas9 fused transcription activator (orange) to activate gene expression. Cas9 nickase (D10A) is a mutant form of the Cas9 nuclease that has a mutation in the RuvC1 nuclease domain, allowing it to cut on a single strand instead of generating a DBS. crRNA, CRISPR RNA; tracrRNA, trans-activating CRISPR RNA; HNH, His-Asn-His endonuclease domain; RuvC, homology domain of UV-sensitive gene product C activity domain, PAM, protospacer adjacent motif; CRISRPi, CRISPR interference; CRISRPa, CRISPR activation; Cas9n, Cas9 nickase.

The evolution of the CRISPR system has also given rise to targeted regulation of gene expression via CRISPR interference (CRISPRi) and CRISPR activation (CRISPRa) techniques. These methods utilize a nuclease-deficient Cas9 (dCas9) to regulate gene expression without permanent genome alterations (Fig. 3). In this context, Kao and Ng (2017) achieved a 94% reduction in expression of the exogenous RFP gene, and their work also demonstrated that down-regulating the endogenous *CrPEPC1* gene, which impacts carbon flux towards lipid production, could effectively increase biomass and lipid accumulation. The findings suggested that CRISPRi-based transcriptional silencing is not only applicable in *C. reinhardtii* but also presents opportunities to improve the productivity, titer, and yield of microalgae-derived products. Following this, the same research group expanded their study to *Chlorella sorokiniana*, proving that both CRISPRi and CRISPRa show high potential as gene-regulating approaches to this microalga (Lin et al., 2022).

In 2018, further progress was made on DNA-free approaches that combine RNPs targeting an endogenous marker gene with RNPs targeting an additional gene of interest, successfully generating dozens of transgenic strains free of foreign DNA in the diatom *P. tricornutum* (Serif et al., 2018). This study confirms the effectiveness of RNP delivery in simultaneously disrupting two counter-selectable endogenous markers, namely the *PtUMPS* and *PtAPT* genes, thereby illustrating the potential for multiplexing to create double-gene knockout strains with efficiencies ranging from 65% to 100% without the need for antibiotic selection. Furthermore, the generation of triple-gene knockout strains was accomplished in a single procedure by introducing six RNP complexes into *P. tricornutum* cells, highlighting the versatility of this approach for application to other difficult-to-transform organisms of biotechnological interest.

The improvement of CRISPR/Cas9 precision technology is still underway. The mutated version of the Cas9 enzyme, known as Cas9 nickase, is designed to induce single-strand breaks rather than double-strand breaks in the target DNA. This strategy significantly reduces off-target effects by creating single-stranded cuts that require cellular DNA repair machinery, primarily HDR, to accurately insert or replace genetic material. Cas9 nickase was reported to successfully introduce mutations into the gene encoding a putative θ-type carbonic anhydrase in the marine diatom *T. pseudonana*, and no mutations were detected at six potential off-target sites (Nawaly et al., 2020).

## CRISPR Toolboxes for Microalgal Gene Editing

**Fig. 4.**
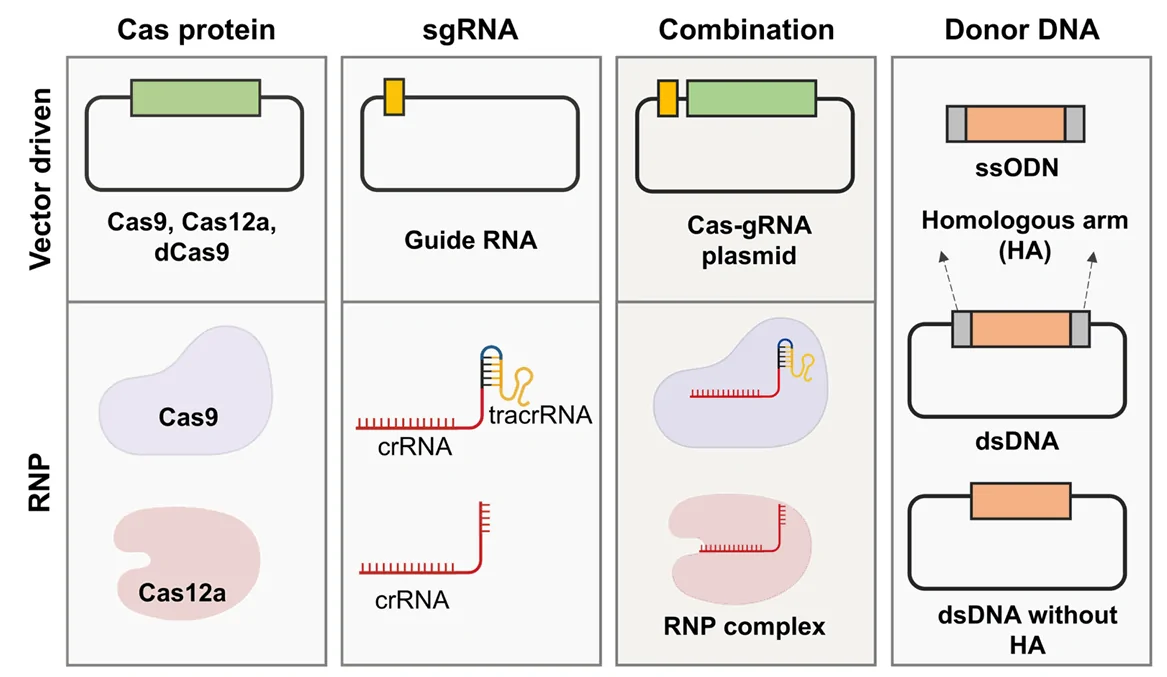
Schematic diagram of approaches and key components for CRISPR/Cas-based gene editing in microalgae. Both ribonucleoprotein (RNP)-based and plasmid vector-based strategies employ Cas proteins and sgRNA to create double-stranded breaks at the target site. The RNP-based approach involves the direct delivery of preassembled Cas proteins and sgRNAs complexes into cells, while the plasmid vector-based approach uses plasmid DNA to encode Cas proteins and sgRNAs, allowing their expression in the host cells. Both methods can incorporate donor DNA fragments, with or without homologous arms, to facilitate homology-directed repair (HDR), enabling precise genome modifications such as point mutations and gene insertions.

The CRISPR/Cas technology has revolutionized genome editing across various organisms and has been extensively studied in microalgae over the past ten years (Table 1). To date, many CRISPR toolboxes tailored for microalgal applications have been developed (Table 2). In addition to these toolboxes, many publicly available protocols have been established, varying depending on the microalgal species, transformation method, and target genomic location. These protocols include a range of techniques, including electroporation, biolistic delivery, and viral-based transformation, each optimized for specific species to enhance editing efficiency and genetic stability (Nymark et al., 2017; Picariello et al., 2020; Wang et al., 2018; Yu et al., 2017). Overall, CRISPR/Cas can be implemented in microalgae through different methods: (1) by introducing a plasmid vector that encodes both the Cas9 protein and the gRNA; (2) by utilizing RNP, which consists of a preassembled Cas9 protein and sgRNA. This section focuses on the key components of CRISPR/Cas used in microalgae and provides insights into the factors that contribute to the successful implementation of the CRISPR/Cas system in these organisms (Fig. 4).

### Plasmid vectors in CRISPR/Cas genome editing

Plasmid vectors are the most traditional strategy for genome editing using CRISPR/Cas. This approach involves the transfection of both the Cas9 and gRNA encoding genes into target cells, leading to the subsequent production of Cas9 protein and gRNA. The plasmid-based method has emerged as the most popular format for CRISPR/Cas gene editing across various organisms, including microalgae, primarily due to its remarkable stability, cost-effectiveness, and ease of preparation (Ran et al., 2013). Moreover, this method allows for simultaneous polygenic editing at multiple genomic sites through the use of different sgRNA designs. Despite these advantages, the plasmid vector system presents several drawbacks, such as delayed responsiveness and the potential induction of immunogenic responses. The efficiency of the plasmid-based method is hindered due to the numerous steps involved in the delivery and processing within the cellular environment. After introduction into a cell, the plasmid must navigate to the nucleus for the transcription of Cas9 mRNA and gRNA. This is followed by the translation of the Cas9 protein from the processed mRNA, and finally the formation of the Cas9/gRNA complex, which is tasked with cutting chromosomal DNA post-nuclear translocation. Of greater concern, the prolonged expression of Cas9 proteins can lead to increased off-target effects and a higher chance of uncontrolled plasmid sequence integration (Tsai & Joung, 2016). To maximize the efficacy of the integrative plasmid-based method, it is essential to optimize several essential components, including the DNA cassettes for expressing the Cas9 gene and gRNA, selection markers such as antibiotic resistance genes or fluorescent proteins, as well as additional functional elements.

**Cas protein expression:** When the first CRISPR/Cas9 genome editing was reported in *C. reinhardtii*, researchers encountered challenges related to the cytotoxicity associated with the constitutive expression of Cas9 in this microalga (Jiang et al., 2014). However, it is worth noting that plasmid-driven expression of Cas9 has been reported to work well in *P. tricornutum*, *N. oceanica*, or *T. pseudonana*, enabling efficient targeted genome editing (Hopes et al., 2016; Nymark et al., 2016; Wang et al., 2016). The most commonly used Cas9 gene variant in microalgae has been the *Sp*Cas9 from *S. pyogenes* (Table 3). In various studies, the DNA sequences of Cas proteins have been codon-optimized specifically for the target algal species (Table 3). For example, the Cas9 gene derived from *S. pyogenes* or *Staphylococcus aureus* has been codon-optimized for efficient expression in *C. reinhardtii*, successfully facilitating targeted gene disruption for both exogenously provided gene targets and endogenous genes of *C. reinhardtii* (Greiner et al., 2017; Jiang et al., 2014; Jiang and Weeks, 2017; Lee et al., 2022). Most studies involving *P. tricornutum* have used diatom-codon optimized Cas9 in plasmid-based systems (Table 3). On the other hand, *S. pyogenes* Cas9 carrying a human codon bias together with SV40 nuclear localization signal, has demonstrated precise and efficient genome editing in *T. pseudonana* (Görlich et al., 2019; Hopes et al., 2016). A pioneering effort using CRISPR/Cas9 technology for gene manipulation in *Chlorella* employed an *Agrobacterium*-mediated plasmid containing a maize-codon optimized Cas9 fragment. This study successfully edited the omega-3 desaturase (*fad3*) gene in *C. vulgaris* FSP-E, resulting in a 46% increase in lipid productivity compared to the wild type strain (Lin & Ng, 2020). In addition, maize-codon optimized dCas9 has been shown to be effective in regulating gene expression in both *C. reinhardtii* and *C. sorokiniana* (Kao & Ng, 2017; Lin et al., 2022). However, there is currently no clear evidence to determine whether codon optimization can enhance the efficiency of plasmid-based CRISPR/Cas in microalgae.

Several research groups have used the cauliflower mosaic virus (*CaMV*) 35S constitutive promoters for plasmid-encoded Cas9 in microalgae (Jiang et al., 2014; Jiang & Weeks, 2017; Kim et al., 2021, 2024; Lin & Ng, 2020). However, the expression of Cas9 on a high-copy vector using a strong constitutive promoter led to a negative influence on the generation of mutations. Thus, endogenous and inducible promoters have proven to be more suitable for producing mutants in microalgae (Table 4). In an effort to test the promoter functionality in Cas9 protein expression, researchers employed two plasmid-encoded Cas9 variants (*Sa*Cas9 and *Sp*Cas9) to target disruption of the phytoene synthase-1 (*PSY1*) gene in *C. reinhardtii* (Greiner et al., 2017). They found that exchanging the heat-shock-inducible HSP70A promoter with the constitutively active HSP70A/RBCS2 promoter led to the production of fewer psy1 clones. Overall, while the form of Cas9 variants does not seem to be a critical parameter, the promoters used for the expression of Cas9 play an important role in engineering strategies for microalgae.

**Guide RNA expression:** The design, expression, and delivery of the gRNA components are crucial parameters for successful CRISPR/Cas9 engineering. A common strategy has been to express a chimeric gRNA molecule from a high-copy vector to ensure abundant expression. The RNA polymerase III U6 promoters are a popular choice for sgRNA expression in microalgae (Table 4), aligning with reports from other organisms (Kor et al., 2023). In general, researchers integrate the Cas9 gene and the appropriately designed sgRNA gene into a single plasmid vector, where separate cassettes are used to express each component, to increase the possibility of co-delivery these components (Hopes et al., 2016; Jiang et al., 2014; Kim et al., 2021; Lin & Ng, 2020; Nymark et al., 2016; Russo et al., 2018; Wang et al., 2016, 2021). Alternatively, the use of two separate plasmids has also been documented, with one plasmid housing a codon-optimized Cas9 gene and the other containing a U6-driven sgRNA gene alongside a *PmR* gene for the selection of transformed cells (Greiner et al., 2017). There is currently no clear evidence addressing the effects of using single versus multiple plasmids on delivering CRISPR/Cas components into microalgal cells.

Another strategy to improve the efficiency of plasmid-based Cas9 activity in *C. reinhardtii* involves utilizing a hybrid construct. In this approach, the sgRNA gene is inserted into an artificial intron, which is then placed at a potential intron splice site within the coding region of the Cas9 gene. This strategy anticipates that following transcription and excision of the intron, the sgRNA would be recognized and productively bound by Cas9 generated from the hybrid Cas9/intron sgRNA gene (Jiang & Weeks, 2017). Similar techniques for integrating Cas9 and gRNA into a single expression cassette have been reported for the knockout of the chloroplastic aldolases/rubisco lysine methyltransferase to enhance biomass production in *N. oceanica* under high-light stress (Liang et al., 2024). Additionally, functional transcription of gRNA can be achieved using the endogenous ribosomal subunit bidirectional promoter (pRibi), which drives the dual expression of both Cas9 and sgRNA genes within a single plasmid in *N. oceanica* (Liang et al., 2024; Wang et al., 2021; Wei et al., 2022).

The application of multiple sgRNAs targeting a single gene has proven effective in inducing large deletions in *T. pseudonana* (Hopes et al., 2016). Another research group established a method employing dual sgRNAs to precisely and serially delete large genome fragments from approximately 100 kb to 214 kb from the 30.01 Mb nuclear genome of *N. oceanica* (Wang et al., 2021). In this study, the researchers designed a plasmid vector that incorporates multiple sgRNA sequences along with self-cleaving ribozyme sequences, including hammerhead (HH) and hepatitis delta virus (HDV) ribozymes. During the transcription, the HH and HDV sequences facilitate the self-cleavage of the nascent RNA transcript, resulting in two distinct functional sgRNAs ready for genome editing.

### RNP-based approaches

In contrast to plasmid-based methods that rely on the transcription and translation of CRISPR components within cells, RNP-based approaches involve the assembly of Cas9 RNP complexes from recombinant Cas proteins and *in vitro*-transcribed sgRNAs for direct delivery into target cells. These components can be introduced through various means, including electroporation (Baek et al., 2016; Ferenczi et al., 2017; Nguyen et al., 2020; Shin et al., 2016, 2019), bombardment (Jeon et al., 2021; Kim et al., 2021; Serif et al., 2018), or glass beads (Zadabbas Shahabadi et al., 2023). This process may or may not include a donor DNA template, depending on the desired genetic modification. Direct delivery of Cas9 RNPs offers various advantages, such as enhanced editing efficiency and reduced toxicity from Cas9 expression, while eliminating the laborious cloning process (Shin et al., 2016). In addition, the transient nature of Cas9, which is rapidly degraded by endogenous proteases, minimizes off-target effects and mosaicism, thereby providing a more precise genome editing option (Baek et al., 2016). Since the Cas9 protein does not integrate into the host genome, this strategy is recognized as a non-genetically modified organism (non-GMO) approach, avoiding the GMO debate (Chang et al., 2020). However, RNP-based editing also has some limitations. Compatibility issues between sgRNA and Cas9 protein can significantly hinder successful editing, underscoring the importance of testing their compatibility prior to transformation. In addition, relatively large RNP complexes may pose challenges for efficient transfection, especially in cell types with limited uptake capacity (Patel et al., 2023). These considerations emphasize the need to optimize RNP components to ensure high editing efficiency and minimize potential drawbacks.

**Variations of Cas protein:** The most commonly used Cas variant in microalgae RNP-based methods is *S. pyogenes* Cas9 (*Sp*Cas9). This variant is typically fused to a nuclear localization sequence to enhance its targeting efficiency within the nucleus (Baek et al., 2016; Kang et al., 2020; Shin et al., 2016, 2019; Zadabbas Shahabadi et al., 2023). The selection of a suitable Cas variant is crucial, as different homologs interact with distinct protospacer adjacent motifs (PAMs). For example, *Sp*Cas9 binds to the 5'-NGG-3' PAM sequence, while *Sa*Cas9 (from *S. aureus*) recognizes the 5'-NNGRRT-3' sequence, where R indicates either adenine or guanine (Greiner et al., 2017). Additionally, Cas12a variants, including *Lb*Cas12a (from *Lachnospiraceae bacterium* ND2006), *As*Cas12a (from *Acidaminococcus* sp. BV3L6), and *Fn*Cas12a (from *Francisella novicida*), target the 5'-TTTV-3' PAM sequence. A longer PAM sequence can reduce potential off-target sites in the genome, thereby enhancing the accuracy of CRISPR-based gene editing (Guo et al., 2023). After selecting a suitable Cas protein, researchers can purchase it from various companies such as ToolGen, Inc. (Korea) (Baek et al., 2016; Chang et al., 2020; Shin et al., 2016), Macrogen, Inc. (Korea) (Jeon et al., 2021), Integrated DNA Technologies (IDT, USA) (Nomura et al., 2019), and New England BioLabs (NEB, USA) (Kang et al., 2020), or codon-optimized and expressed in an *E. coli* system with the T7 promoter for efficient transcription and protein production in laboratory settings (Naduthodi et al., 2019; Zadabbas Shahabadi et al., 2023) (Table 2). Cas proteins differ in size, which can affect their efficiency in RNP-based genome editing applications. For instance, *Sp*Cas9, at 163 kDa, is a relatively larger compared to the smaller *Sa*Cas9 at 124 kDa, which has documented high indel mutation rates between 77.7 and 90.1% on targets like *EgGSL2* (Nomura et al., 2019). Likewise, Cas12a (Cpf1) proteins exhibit different sizes: *Lb*Cas12a at 149 kDa, *As*Cas12a at 156 kDa, and *Fn*Cas12a at 151 kDa (Mohanraju et al., 2018). Among these, *Fn*Cas12a has shown the highest efficiency for HDR-based targeted mutagenesis, achieving up to 93% mutants among transformants, while *As*Cas12a has the lowest efficiency in *N. oceanica* IMET1 (Naduthodi et al., 2019). Such findings indicate that smaller Cas proteins may confer benefits in specific applications, as RNP-based editing efficiency is influenced by cell surface structures and the physicochemical properties of RNP molecules. In summary, the choice of Cas protein variant, together with a well-designed sgRNA, is essential for the optimization of RNP-based gene editing, significantly impacting the overall success of the approach.

**sgRNA:** To successfully target a specific gene, Cas proteins depend on sgRNAs to guide them to the desired DNA sequence. In the case of Cas9, the sgRNA is composed of a custom-designed crRNA fused to a tracrRNA, typically around 120 nucleotides in length. In contrast, Cas12a uses shorter crRNA molecules, approximately 43 nucleotides long, to guide its activity to the target site. Similar to Cas proteins, sgRNAs can be obtained from specialized commercial providers. These commercially available sgRNAs offer high purity, consistency, and convenience, making them particularly suitable for time-critical or high-throughput applications. Alternatively, researchers can produce *in vitro*-transcribed sgRNAs in the laboratory using various kits, such as the MEGAshortscript^TM^ T7 Kit (Ambion, USA) (Baek et al., 2016), HiScribe T7 RNA Kit (New England Biolabs, USA) (Naduthodi et al., 2019), CUGA7 gRNA Synthesis Kit (Nippon Gene, Japan) (Picariello et al., 2020), and EnGen® sgRNA Synthesis Kit (New England Biolabs, USA) (Freudenberg et al., 2022) (Table 2). These methods are often more cost-effective and scalable, allowing researchers to customize sgRNAs on demand for large-scale or multi-target applications.

For efficient gene editing, the general process involves designing and testing multiple sgRNAs targeting a single gene to evaluate their cleavage efficiency, often using in vitro cleavage assays (Freudenberg et al., 2022). The sgRNA demonstrating the highest cleavage efficiency is selected for transformation. For instance, Shin et al. (2016) tested two sgRNAs against *MAA7* gene and found that the first failed to cleave the target, while the second successfully cleaved the genomic DNA target into two fragments. Building on this foundation, several studies have shown that combining multiple RNP complexes containing two or more sgRNAs targeting the same gene can further enhance editing efficiency (Chang et al., 2020; Naduthodi et al., 2019; Serif et al., 2018). For example, editing efficiencies of 65–100% were achieved using two sgRNAs per target for gene editing of *UMPS*, *APT*, *Aureo1a* in *P. tricornutum* (Serif et al., 2018). Similarly, in *N. oceanica*, Cas9-mediated editing with two sgRNAs targeting the *NR* gene resulted in efficiencies ranging from 34% to 71%. In contrast, Cas12a achieved a broader range of editing efficiencies from 3% to 93% mutant colonies by using three random crRNAs per target (Naduthodi et al., 2019).

In addition to the utilization of multiple sgRNAs, careful optimization of both sgRNA and Cas9 concentrations significantly influences the efficiency of gene editing by ensuring the formation of functional RNP complexes (Kouranova et al., 2016). The ratio of sgRNA to Cas9 protein (sgRNA∶Cas9) used for RNP complex formation can vary based on the experiment setup and the desired outcomes. For example, (Shin et al., 2016) maintained a 4:3 mass ratio of sgRNA (40 µg) to Cas9 protein (30 μg), while (Kang et al., 2020) used a ratio of 13.3 µg sgRNA to 10 µg Cas9. (Baek et al., 2016) reported a commonly used ratio of 7:10, preassembling 200 µg of Cas9 with 140 µg of sgRNA. Additionally, Shin et al. (2019) and Song et al. (2020) employed a ratio of 100 µg Cas9 to 70 µg sgRNA. Equimolar ratios, such as 10 µM sgRNA and 10 µM Cas9, are also commonly used to ensure balanced RNP assembly (Naduthodi et al., 2019). Therefore, optimizing the sgRNA/Cas9 ratio is essential for ensuring efficient and reliable gene editing outcomes across diverse experimental systems.

**DNA repair templates:** In RNP-based genome editing, the choice of donor DNA template for HDR can be either single-stranded oligonucleotides (ssODNs) or dsDNA constructs. ssODNs are short, single-stranded molecules that typically carry small genetic modifications, such as point mutations or insertions. They are favored for their smaller size and easier delivery into cells (Akella et al., 2021; Nomura et al., 2019). In contrast, dsDNA donor templates, which can be linear or plasmid-based, provide longer homology arms and are generally more efficient at promoting HDR, especially in dividing cells. Donor templates in genome editing can incorporate a selection gene cassette, which encodes for a selectable marker that facilitates the identification and selection of successfully edited cells (Nguyen et al., 2020; Picariello et al., 2020). This selection gene cassette is often inserted into the donor template to simplify the process of identifying cells that have undergone successful genome modification. Both ssODNs and dsDNA templates are introduced alongside the CRISPR/Cas9 RNP complexes, and the selection between the two depends on the size and complexity of the desired genetic alteration, as well as the efficiency of the delivery systems employed (Freudenberg et al., 2022). In *C. reinhardtii*, researcher has shown that short (80 nucleotides) single-stranded DNA (ssDNA) fragments with protected ends, which are homologous to specific sites in the target genes, can effectively stimulate homologous gene or nucleotide replacement using plasmid-based Cas9 (Jiang & Weeks, 2017). Moreover, co-delivery of CRISPR/Cas12 RNPs with ssDNA repair templates has been shown to result in precise DNA replacement with up to ~10% efficiency in *C. reinhardtii* (Ferenczi et al., 2017).

## Challenges and Solutions in Utilizing CRISPR/Cas System in Microalgae

Despite extensive studies on CRISPR/Cas9-mediated genome editing, several challenges must be addressed when implementing this system in microalgae. The limited transformation efficiency in these organisms arises from several factors: (1) the presence of physical barriers, such as the cell wall and cytoplasmic membrane of microalgae, which act as protective layers preventing the penetration of foreign substances; (2) the absence of suitable selection markers; and (3) off-target effects and low efficiency of HDR in certain microalgae strains.

### Delivery

One of the main challenges in applying the CRISPR/Cas9 system to microalgae is effectively delivering its components, including the Cas9 protein and gRNA, into cells. Delivery formats for the CRISPR system in other organisms include DNA, mRNA, and protein (Kouranova et al., 2016). Among these, DNA plasmids are the most commonly used source for Cas9 nuclease in microalgal genome editing. However, the multistep process from plasmid delivery to mRNA transcription and formation of the mature Cas/gRNA complex can be inefficient. In contrast, delivering Cas9 as mRNA allows bypassing transcription, as it only requires entry into the cytoplasm for translation. However, the unstable nature of mRNA presents challenges for secure delivery and rapid expression in the physiological environment. Currently, there are no reports on mRNA delivery of Cas9 in microalgae. Lastly, the RNP complex represents the simplest and most efficient means of delivery with higher gene editing efficiency. However, the delivery of the large RNP complex can be complicated for microalgae with robust cell walls.

Various physical methods for directly delivering CRISPR components into microalgal cells have been used, including electroporation and biolistic bombardment. Electroporation uses optimized electrical pulses to transiently disrupt the cell membrane, creating temporary pores for CRISPR components entry. This technique is favored for its broad applicability across diverse cell types. Thus, electroporation remains a preferred method for delivering the Cas9 protein in many microalgae, yielding mutagenesis efficiency ranging from 0.14% to 95% in *C. reinhardtii*, 0.1% to 78% in *Nannochloropsis*, approximately 50% in *Chlorella*, and 77.2% to 94.5% in *E. gracilis* (Table 1). It is worth noting that the reported mutagenesis efficiency reflects the percentage of mutants confirmed by genotyping out of the total genotyped mutants, not accounting for the size of the initial population. On the other hand, bombardment appears to be more suitable for diatoms such as *P. triconutum* and *T. pseudonana*, achieving a maximum mutagenesis efficiency of 100% for *P. triconutum* and 39% for *T. pseudonana*. However, this method may cause physical damage to cells and potentially result in large rearrangements of the host genome. Using glass beads for CRISPR component delivery has shown varied results in *C. reinhardtii*, showing lower efficiency for the RNP complexes compared to plasmid vectors, with 14.81% versus 94%, respectively. Notably, the combination of Cas9 RNP and cell-penetrating peptides, such as pVEC, has induced gene editing efficiencies ranging from 8.41% to 46.56% in *C. reinhardtii*. Researchers have also investigated bacterial conjugation for delivering CRISPR/Cas9 plasmids in *P. tricornutum* (Daboussi et al., 2014). Although *Agrobacterium*-mediated transformation of CRISPR has not been reported for microalgae, Lin et al. (2022) employed a plasmid containing the left and right borders, essential for homologous flanks in the transfer DNA (T-DNA) binary system, followed by electroporation to apply CRISPR in *C. sorokiniana*.

Electroporation of RNPs combined with autolysin treatment significantly improved gene editing frequencies, as demonstrated in CRISPR-based targeted insertional mutagenesis (TIM) (Picariello et al., 2020). TIM achieved mutation efficiencies ranging from 40% to 95%, highlighting the critical role of cell wall removal for successful RNP delivery and efficient genome editing in *C. reinhardtii*. Another study addressed this challenge by using Alcalase treatment to selectively weaken the cell wall of *C. sorokiniana* (Kim et al., 2024). Alcalase-treated cells exhibited a permeability of 26% in intact cells, successfully transferring TAMRA-labeled DNA (789 bp) into *C. sorokiniana* cells, although gene expression remains a barrier to gene editing in this species.

In addition, a stable line expressing Cas9, referred to as NgCas9+, has been established in *N. gaditana*. This NgCas9+ line enables the co-electroporation of *in vitro*-synthesized gRNA along with an antibiotic selection marker, facilitating efficient targeted integration with up to 80% of colonies containing an insertion at the desired locus (Ajjawi et al., 2017; Verruto et al., 2018). It has been observed that raising the recovery temperature to 33°C enhances targeting events in both ZFNs and Cas9 plasmid-encoded nuclease systems, although it does not influence the efficiency of recombinant Cas9/gRNA RNPs. Moreover, a heat shock treatment for 30 min prior to transformation is thought to induce physiological changes that may benefit the activity of Cas9/gRNA and help with DNA repair, DNA integration, and HR, which necessitate further research (Greiner et al., 2017).

### Selection strategies for CRISPR-edited microalgal mutants

The implementation of effective selection strategies is crucial for maximizing the chances of achieving target mutations in CRISPR-edited microalgal mutants. A common approach involves the utilization of endogenous selectable markers. To date, various endogenous selective markers have been used for gene editing in these organisms, including nitrate reductase, adenine phosphoribosyl transferase, peptidylprolyl isomerase, tryptophan synthase beta subunit, orotidine 5′-phosphate decarboxylase, protoporphyrinogen IX oxidase, and spermidine synthase (Akella et al., 2021; Freudenberg et al., 2022; Jiang & Weeks, 2017; Serif et al., 2018; Shin et al., 2016; Wang et al., 2016). Co-targeting a gene of interest alongside an endogenous selective marker can significantly improve the frequency of intended gene modifications in microalgae without integration of transgenes. However, relying solely on phenotypic changes, such as visible alterations, to select genome-edited cells is often inefficient and can be laborious and time-consuming (Baek et al., 2016; Chang et al., 2020; Shin et al., 2016). To address this challenge, pre-selection using antibiotic resistance has been employed, significantly boosting selection efficiency to as high as 90% (Greiner et al., 2017; Picariello et al., 2020; Shin et al., 2016). Furthermore, incorporating reporter genes, such as luciferase or mVenus, directly into the targeted gene site facilitates straightforward identification of mutants (Freudenberg et al., 2022).

To comply with biocontainment requirements necessary for scaling up production in open ponds, modified algae must be free of antibiotic markers. Recent studies have successfully achieved this by using CRISPR-mediated mutants with selection strategies based on the use of episomal vectors. It has been reported that episomes containing a fragment of the bleomycin antibiotic coding sequence, combined with the yeast centromeric CEN6-ARSH4-HIS3 sequence, enable episome maintenance in *P. tricornutum* (Karas et al., 2015). This protocol was adapted to develop an episomal CRISPR/Cas9 system using the CEN/ARS6 region from *S. cerevisiae*, allowing the generation of markerless mutants for *N. oceanica* (Poliner et al., 2018). In this approach, mutants were initially selected based on their resistance to the antibiotic Hygromycin. Once the desired mutation at the target site was identified, the antibiotic selective pressure was removed. As a result, the researchers successfully disrupted the *NR* gene and subsequently eliminated the CRISPR episome, thereby generating non-transgenic, marker-free knockout mutants in this marine alga. Additionally, Verruto et al. (2018) achieved the generation of markerless *N. gaditana* mutants by combining CRISPR/Cas9 gene editing with inducible Cre-mediated recombination. This method allows for the removal and recycling of antibiotic selection markers, thus facilitating the stacking of up to seven gene knockouts in the *N. gaditana* host strain.

### Off-target effects and low efficiency of HDR

While high specificity is often considered the hallmark of CRISPR technology, the potential for unintended genome alterations reduces confidence in using this technique for precision genetic engineering, especially in commercially relevant species. This concern is particularly pronounced in microalgae, where unique genomic structures and the presence of repetitive sequences can increase the chances of off-target events occurring. Several factors contribute to the accuracy of targeting, including the design of gRNAs, type of nuclease used, and the recognition sequences associated with PAM. Various tools are available for designing target-specific gRNAs and predicting potential off-target sites in microalgae, including Cas-OFFinder (Baek et al., 2016; Ferenczi et al., 2017; Görlich et al., 2019; Nawaly et al., 2020; Shin et al., 2016; Wang et al., 2021), CHOPCHOP (Zadabbas Shahabadi et al., 2023), CRISPRdirect (Picariello et al., 2020; Yoneda et al., 2023), CRISPOR (Serif et al., 2018), and Benchling (Xie et al., 2023). Studies have indicated that the occurrence of off-target mutations correlates with the abundance of the Cas9/sgRNA complex, suggesting that transient expression of these components may reduce the probability of such effects compared to stable genomic integration. For example, Shin et al. (2016) reported no off-target mutations detected within 333 potential sites related to the *CpSRP43* gene when utilizing a Cas9 RNP system to knockout the gene in *C. reinhardtii*. This finding implies that minimal off-target events may result from degradation or dilution of Cas9 RNPs shortly following electroporation. In silico predictions of potential off-target sites, combined with whole genome sequencing or a mix of PCR and Sanger sequencing of mutants, allow for the detection of off-target events in various microalgal species. While whole genome sequencing provides a comprehensive assessment, its effectiveness can be limited by the high rates of spontaneous mutations and the polymorphic characteristics of diatom genomes, particularly when identifying small indels (Rastogi et al., 2020). A proposed solution to mitigate these off-target effects involves targeting both sense and antisense DNA strands using a pair of sgRNAs. By employing Cas9 nickase with two sgRNAs to create double nicks in the target DNA, Nawaly et al. (2020) successfully introduced precise and relatively short biallelic indels to the genome of *T. pseudonana*, with no off-target events detected.

Low efficiencies of HDR present significant challenges in achieving precise targeted knockin of desired gene fragments in microalgae. Despite the implementation of various optimization strategies, such as adjusting the concentrations of DNA fragments, lengths of HDR cassettes, and optimizing the transformation conditions, the success rate of HDR remains low in *C. reinhardtii* (Plecenikova et al., 2013). Conversely, *Nannochloropsis* species exhibit substantially higher HR activity (Kilian et al., 2011). The co-delivery of Cas9/gRNA RNP complexes along with dsDNA repair templates has proven to enhance HDR at the target site (Naduthodi et al., 2019). In addition, recent studies indicate that transgene with 1,000-bp homology arms successfully induce HDR, achieving a targeted knockin efficiency of 15% in *C. reinhardtii* (Zadabbas Shahabadi et al., 2023). Furthermore, research by Angstenberger et al. (2020) highlighted that synchronizing *C. reinhardtii* cultures at 28°C during the light phase and 18°C during the dark phase can significantly enhance HDR efficiencies, particularly at 12 h post-illumination initiation.

### Limitations of CRISPR/Cas technology in microalgae

CRISPR/Cas technology is a powerful tool for the genetic manipulation of microalgae to enhance their performance. However, exploiting its full potential is significantly hindered by many limitations in microalgal genomic data, database quality, and research scalability. Although an estimated 200,000 to 800,000 species of microalgae exist, only about 50,000 have been described, with only a small fraction having their genomes fully sequenced and characterized (Venkatesan et al., 2015). This limited genetic information constrains our understanding of microalgal metabolism and cellular pathways that could be leveraged for industrial application. For instance, many microalgae species possess unique metabolic traits that are potentially valuable for biotechnological application. However, these species remain under-researched due to a lack of comprehensive genomic data, which hinders both research and commercialization efforts (Khan et al., 2018). Furthermore, the genomic databases that do exist often lack comprehensive functional annotations, limiting their usability in identifying gene functions and their applications in biotechnology (Aoki et al., 2016). Current databases often suffer from inconsistencies, redundancies, and inadequate management, which undermine the reliability of the extracted information. For effective use, comprehensive and well-maintained databases that provide not only genomic sequences but also functional annotations, gene co-expression data, and metabolic pathways are needed.

Moreover, the prominence of model species such as *C. reinhardtii* and *N. oceanica* overshadows other potentially valuable species that have yet to be adequately explored. This imbalance in strain selection arises primarily from the availability of genomic information and the strain’s potential for specific applications, such as lipid production, which has received considerable attention compared to other fields. This focus limits the research diversity needed to explore new applications and optimize existing ones in microalgal biotechnology. Given the constraints of funding and time, it is indeed difficult to argue for prioritizing microalgal research over other microbial studies. Nonetheless, certain microalgal species possess unique industrial potential that warrants further investigation. For instance, *P. tricornutum* is a promising candidate due to its feasibility in producing fucoxanthin, EPA, and DHA at commercial scale. In Europe, this alga is currently cultivated by several companies, achieving an annual production of 4 tons of dry biomass (Araújo et al., 2021). Among marine diatoms, *P. tricornutum* has emerged as an experimental model with the most extensive genetic toolbox available. Advances in genetic tools could further unlock the industrial potential of this strain. Another notable candidate is the species *Tetraselmis* (Chlorophyta), a green marine alga that has been selected and studied for its utility in biodiesel production in open culture systems and bubble column photobioreactors (Kim et al., 2017). For industrial-scale biofuel production, genetic engineering approaches are needed to generate mutant strains that can enhance lipid production without compromising biomass yield. Similarly, *Dunaliella salina*, known for its high carotenoid production, also produces compounds that are active against several strains of bacteria and fungi (Khan et al., 2018). Despite its promise, the industrial cultivation of this alga is not without challenges. Improvements in biomass yield and β-carotene production may benefit from advanced genome editing strategies aimed at increasing metabolite yields. Further research on these species will help expand the field and improve the applicability of CRISPR/Cas9 in microalgal biotechnology. Another limiting factor affecting the industry is the scale of research conducted on microalgae. The disparity between laboratory-scale experiments and industrial applicability often leads to findings that do not translate effectively from controlled settings to larger scales (Rozenberg et al., 2024). The focus on a limited number of microalgae species exacerbates this issue, as only 15 genera dominate research, representing a mere 0.02–0.05% of the total estimated diversity. Additionally, the pressures of achieving economically viable production processes result in lower investment in novel species research, which could otherwise expand the field.

## Applications of CRISPR/Cas in Microalgae

The development of CRISPR technology has revolutionized genetic engineering in microalgae, providing precise and efficient tools for targeted genome editing. This advancement has significantly facilitated the optimization of metabolic pathways and the functional exploration of microalgal genomes. Despite encountering certain challenges, the CRISPR/Cas system has been successfully applied to develop microalgal strains for various industrial applications.

### Biofuel

In biofuel production, CRISPR-mediated metabolic engineering has effectively enhanced lipid accumulation in microalgae. The first successful attempt to use CRISPR/Cas9 to engineer an industrial microalgal strain for improved lipid production was demonstrated in *N. gaditana* in 2017 (Ajjawi et al., 2017). Researchers employed a CRISPR/Cas9-based reverse genetics pipeline to target transcription factors identified through RNA-seq analysis conducted during nitrogen deprivation. This study demonstrates the role of these transcription factors as negative regulators of lipid accumulation. Among the 20 transcription factors screened, the disruption of a homolog to the fungal Zn(ii)2Cys6 genes resulted in a dramatic improvement in lipid partitioning, with lipid content increasing from 20% in the wild type to 40–55% in the mutants under nutrient-rich conditions (Ajjawi et al., 2017). Furthermore, knocking out the *C. reinhardtii* phospholipase A2 gene resulted in a higher diacylglycerol pool and increased triacylglycerol accumulation, enhancing overall lipid productivity by 64.25% without significant changes in cell growth (Shin et al., 2019). In a separate study, the targeted knockout of a gene involved in fatty acid degradation in this alga resulted in a mutant strain exhibiting a total lipid accumulation of 28% of dried biomass. This modification was accompanied by a shift in fatty acid composition, notably a 27.2% increase in the C18:1 proportion (Nguyen et al., 2020). Additionally, the functional knockout of the acyl-CoA binding protein (ACBP) affected genes involved in acyl-CoA utilization, including fatty acid synthesis, β-oxidation, and autophagy, particularly during the period when cells rely on endogenous carbon stores. Knocking out of this gene also resulted in inhibition of triacylglycerols (TAGs) lipolysis and was associated with the inhibition of lipid droplets/TAGs degradation observed in other experiments (Leyland et al., 2024). While research has indeed shown the potential of microalgae as a renewable energy source, the transition from laboratory findings to real-world applications has yet to be feasible. Most existing studies have focused on improving microalgal lipid yield and biofuel properties. However, the high costs associated with production processes remain the main barrier to commercial viability of microalgal biofuels. The estimated cost of producing algal biomass for biofuel ranges from $0.50 to $2.26 per kg in open ponds and from $4.80 to $12.60 per kg in photobioreactors, which cannot compete with the low price of fossil fuels (Kang et al., 2015). In addition, downstream processing, including biomass harvesting and dehydration, along with lipid extraction, can account for more than 50% of production costs (Mallick et al., 2016). There is no doubt that strategic advances in cultivation and harvesting technologies are required to improve cost efficiency and overall profitability. In the context of this review, it is worth mentioning that genetic engineering can reduce the cost of microalgal biofuels by 15–20% (Jagadevan et al., 2018). For most microalgae, high biomass does not necessarily equate to high lipid productivity, and high lipid content can actually result in lower biomass productivity. Therefore, one should take both lipid content and biomass yield into account when developing algal strains for biofuel production through genetic modification.

### Photosynthesis and pigments

Modification using CRISPR has also emerged as a powerful tool for improving photosynthetic efficiency and pigment production. The original concept of employing microalgae in industry is to produce valuable compounds while acting as efficient carbon sinks by fixing atmospheric CO_2_ during photosynthesis. For industrial applications, microalgae must achieve growth rates competitive with established bacterial and yeast systems. In accordance, early studies using CRISPR/Cas9 in microalgae targeted genes that regulate light utilization and carbon fixation. One notable advancement is the application of a DNA-free CRISPR/Cas9 method for the one-step transformation of *C. reinhardtii*, which enabled the sequential knockout of the *CpFTSY* and *ZEP* genes (Baek et al., 2016). The knockout of *CpFTSY* gene reduced the size of chlorophyl antennae, allowing better light penetration in dense cultures. Such approach is essential for maximizing biomass production in large-scale cultivation systems. Furthermore, the modification of *ZEP* gene resulted in a strain that exhibits a significant increase in zeaxanthin production without affecting photosynthesis. Since many pigments associated with the photosynthetic system, such as carotenoids, astaxanthin, lutein, phycoerythrin, and phycobiliproteins, are also valuable for human use, targeting the photosynthetic system in microalgae not only for the improvement of light usage, but also for the enhancement of pigment yields for industrial applications. In another study, Cas9 RNPs were used to generate the chlorophyll synthase loss-of-function mutants (Δchs1) in *Porphyridium purpureum*. These mutants showed increased phycoerythrin contents compared to controls, regardless of the light source, while their growth remained unaffected (Jeon et al., 2021). Additionally, the Cas9 RNP-mediated knockout of the ADP-glucose pyrophosphorylase (*AGP*) gene in *Chlamydomonas zep* mutants lacking zeaxanthin epoxidase function resulted in a 1.75-fold increase in oil productivity while maintaining macular pigment production (Song et al., 2022). Both of these studies have generated microalgal mutants with enhanced bio-product production and sustained photosynthetic efficiency, making them promising candidates for industrial application. Importantly, these studies highlight the effectiveness of CRISPR RNP techniques in generating markerless mutants in microalgae, a critical advantage for regulatory approval and marketability.

### Stress tolerance

Regarding stress tolerance and environmental adaptation, gene editing technologies have shown promise in improving the resilience of microalgae to both biotic and abiotic stressors. Specifically, Cas9-mediated knockouts of key cytokinin (CK) biosynthetic genes, such as Lonely Guy (*LOG*) and isopentenyl transferase (*IPT*), have revealed that CK-deficient mutants exhibit a diminished ability to induce plant defense responses when compared to wild type strain (Sandor et al., 2024). In addition, environmental factors, including phosphate starvation, were found to enhance the efficiency of gene editing in *C. reinhardtii* by 22%. Under such conditions, co-editing experiments involving *PSR1* (a transcriptional factor that modulates phosphate starvation response) and *CpFTSY* (a chloroplast signal recognition particle receptor protein) demonstrated the feasibility of DNA-free multiplex gene editing on genes that lack a selectable phenotype (Battarra et al., 2024). This approach has the potential to overcome the limitations associated with antibiotic resistance markers, while enabling the generation of transgene-free *C. reinhardtii* strains for both fundamental research and biotechnological applications.

### Biopharmaceuticals and bioactive molecules production

CRISPR technology also has important applications in the field of biopharmaceuticals and the production of bioactive molecules. For example, CRISPR/Cas9 RNPs have been used to create double-gene knockout mutants targeting the lycopene epsilon cyclase (*LCYE*) gene in *C. reinhardtii* zep mutants. This effectively blocks α-carotene biosynthesis and increases zeaxanthin accumulation by 60% (Song et al., 2020). In a separate study targeting the same *LCYE* gene in *C. reinhardtii*, the researchers achieved astaxanthin accumulation of up to 1.8 ± 0.6 mg/L in the parental strain UVM4, a UV-mutated strain deficient in cell wall (Kneip, 2024). In addition, the targeted knockout of the hydroxy pyruvate reductase 1 (*CrHPR1*) gene in this alga resulted in a significant increase in glycolate production, reaching levels of 280.1 mg^-1^ L^-1^ OD_750_^-1^, with a maximum yield of 2.1 g/L under optimized mixotrophic conditions (Song et al., 2020).

## Perspectives and Future Directions

### Utilization of high-fidelity nucleases

Recent advancements in CRISPR-based gene editing technologies have significant implications for the field of microalgal biotechnology. Researchers have employed various strategies to refine the specificity of commonly used Cas proteins in other organisms. Among these approaches, techniques such as directed evolution, structure- or function-guided protein engineering, or combinations of both have been used for engineering high-fidelity (HiFi) Cas proteins and representative variants. There are notable HiFi variants such as HiFi Cas9, xCas9, SpartaCas, and efSaCas9 (enhance-fidelity *Sa*Cas9), each being distinguished for their reduced off-target effects. Additionally, engineered Cas9 variants such as KKH *Sa*Cas9, which is smaller than *Sp*Cas9 (Ran et al., 2013), could be good candidates due to their potentially higher efficiency in penetration through the microalgal cell wall. Currently, only few variants of Cas proteins have been studied in microalgae. Apart from *Sp*Cas9 variants, other Cas proteins such as *Sa*Cas9 which possess different PAM requirements are increasingly adopted in microalgal gene editing applications due to its high activity in eukaryotic systems (Greiner et al., 2017; Lee et al., 2022; Nomura et al., 2019). Current trends indicate a focused effort to identify and develop HiFi nucleases tailored for microalgal applications. The integration of such advanced nuclease variants will substantially elevate the editing efficiencies in microalgae, facilitating further development in biotechnological areas such as biofuel production and metabolic pathway engineering.

### Sequencing of CRISPR-edited microalgal mutants

Genome editing process, particularly those utilizing CRISPR technology, can affect unintended loci if there is a sequence match between the designed gRNAs and the genome. Hence, the analysis of potential off-target sites through computational analysis is crucial for avoiding possible adverse effects in target cells. Currently, whole genome sequencing of CRISPR-edited clones via next-generation sequencing (NGS) platforms represents the most efficient way to discover off-target editing in many organisms (Guo et al., 2023). NGS technologies not only facilitate the detection of off-target outcomes but also enable the identification of allelic changes in CRISPR-edited mutants. Given the decreasing costs of sequencing technologies and advancements in methodologies, whole genome sequencing of microalgae has become more feasible with many laboratories considering it a practical option for their research. On the other hand, it remains possible to evaluate off-target edits by sequencing genomic regions that are likely to exhibit the highest probability of off-target effects detected by computational analysis. Specifically, validation can be achieved by amplifying the relevant target regions and sequencing the purified fragments via Sanger sequencing. Since Sanger sequencing typically works best with amplicons of shorter length, ideally 500 bp, while lengths above 800 bp may encounter challenges in data fidelity, significance of maintaining an appropriate length cannot be understated.

### Using CRISPR for genomic targeting of other activities

While traditionally used for modifying genomic sequences, CRISPR technology has evolved to enable diverse applications beyond gene editing. As mentioned earlier, CRISPRi and CRISPRa have successfully achieved targeted regulation of gene expression in *C. reinhardtii* and *C. sorokiniana*. Compared to conventional approaches, CRISPR-mediated gene regulation provides precise and stable regulation of gene expression. Another approach involves using CRISPR to construct minimal genomes, which are smaller collections of genes necessary for a cell to sustain life under specific conditions. This reduction in genetic complexity enhances predictability in synthetic biology applications. Researchers have demonstrated that the targeted removal of large, non-essential genomic regions in *N. oceanica* does not compromise key traits of the organism. This approach allows for the development of a minimal genome for this alga, positioning it as a chassis strain for customized CO_2_-driven industrial production (Wang et al., 2021). CRISPR/Cas technology has been integrated into the field of synthetic biology, with its applications continuously expanding. In addition to the potential applications discussed above, researchers can even use CRISPR/Cas to create fully synthetic microalgal cells with unprecedented functionality. In the future, the integration of CRISPR with emerging technologies, such as machine learning and high-throughput genome sequencing, is expected to further enhance experimental efficiency, predictive modeling, and design precision in genetic engineering.

### The two-sided coin of CRISPR technology

The rapid advancement of CRISPR/Cas technology has provided researchers with powerful tools to redesign the genomes of microalgae, resulting in improved traits that bridge the gap between laboratory research and practical applications. CRISPR/Cas has made significant contributions to accelerating the industrialization of microalgae. However, researchers must carefully assess both the benefits and risks of this technology. One of the main advantages of CRISPR/Cas is the ability to achieve markerless genome modifications that can technically be classified as non-genetically modified organism (non-GMOs) (Ahmad et al., 2023). However, the precision of CRISPR does not guarantee that engineered traits will function as intended in natural ecosystems. Moreover, many genetically modified microalgae used for industrial purposes are not native to their cultivation regions. These strains are often selected for their fast growth and resilience to maximize biomass yield. Therefore, there is a risk that CRISPR-edited species could outcompete native species or transfer their engineered traits to wild relatives, potentially leading to ecological homogenization and loss of local biodiversity. While closed bioreactors provide controlled growth conditions that limit ecological risks, their high capital and operational costs make them less viable for low-value products such as biofuels. In contrast, microalga cultivation using wastewater in open systems, such as tanks or ponds, is a more viable approach for this outcome (Pittman et al., 2011). However, this method has the potential for genetically modified microalgae to escape into the environment. This concern highlights the importance of carefully balancing cost-effective production with potential ecological threats.

The hesitancy of the industrial biotechnology to adopt genetically modified microalgae is due to insufficient information and tools to assess their behavior and risks in large-scale production. Adding to this complexity, current regulations on GMOs vary widely across the globe, and guidelines for genome editing techniques like CRISPR/Cas9 remain inconsistent. For example, the U.S. Department of Agriculture (USDA) decided not to regulate a mushroom modified using CRISPR/Cas9, reasoning that the resulting traits were consistent with natural genetic variation and could be considered non-GMO (Waltz, 2016). While the decision facilitates commercialization, critics argue the approach does not adequately addresses safety concerns or the potential long-term ecological impacts of CRISPR-edited organisms. This highlights the need for globally consistent regulations and guidelines to govern the cultivation, commercialization, and monitoring of CRISPR-edited microalgae, as well as other GMOs. Such regulations are essential to maintain ecological integrity, promote public trust, and support the responsible use of CRISPR technology for industrial applications.

## Conclusion

This review emphasizes the use of CRISPR/Cas systems in microalgae, highlighting their potential for precise genome editing and the advancement of microalgal biotechnology. While these applications present numerous opportunities for enhancing productivity and metabolic engineering, several challenges persist. These include inefficiencies in transformation methods, interspecies genetic variation, and concerns regarding off-target effects. Researchers are making efforts to address these hurdles by developing tailored strategies and optimized CRISPR components, such as Cas9, Cas12a, dCas9, and Cas9 nickase, alongside emerging CRISPR-based approaches. This progress is unlocking the full potential of microalgal applications in the production of biofuels, pharmaceuticals, and other valuable biomolecules. Furthermore, this review highlights the importance of improving our understanding of the genetic mechanisms involved to enable large-scale utility of CRISPR/Cas technology in microalgal synthetic biology. The integration of high-fidelity nucleases and systematic sequencing of CRISPR-edited microalgal mutants will further pave the way for increased precision and broader applications of genomic targeting. Collectively, these advances set the stage for future progress in biotechnological applications of microalgae.

## Figures and Tables

**Table 1.** Genome editing using CRISPR/Cas system in microalgae

Algal strain	CRISPR/Cas system	Strategy		Targeted genes	Selection Marker	Products	Mutagenesis efficiency (%)^⸶^	Targeted mutagenesis frequency^⸷^	References
		Approaches	Delivery method						
*Chlamydomonas reinhardtii*	Cas9	Vector driven	Electroporation	*FKB12*	Rapamycin	First successful transient expression of Cas9 and sgRNA genes in *C. reinhardtii*	0.0014	2 × 10^-8^	Jiang et al. (2014)
	Cas9	RNP	Electroporation	*MAA7, CpSRP43, ChlM*	Auxotrophic selection using 5-FI	Visible auxotrophic colonies with mutations targeted at the Cas9 cut sites	40% (*MAA7*), 1.4% (*CpSRP43*), and 0.17% (*ChlM*)	8.9 × 10^-8^ (*MAA7*), 3.3 × 10^-8^ (*CpSRP43*), and 5 × 10^-8^ (*ChlM*)	Shin et al. (2016)
	Cas9	RNP	Electroporation	*CpFTSY, ZEP*	Based on the coloration of the cells	High zeaxanthin-producing mutants with improved photosynthetic productivity	0.46 to 0.56%	*Not mention*	Baek et al. (2016)
	CRISPRi	Vector driven	Glass beads	*PEPC1*	Hygromycin and paromomycin	A 74.4% increase in lipid content and a 94.2% enhancement in lipid productivity	0.94	*Not mention*	Kao & Ng (2017)
	Cas9	Vector driven/RNP	Electroporation	*COP1/2, COP3, COP4, COP5, PHOT, UVR8, VGCC, MAT3, aCRY, PSY1*	Paromomycin	Protocols for the rapid isolation of non-selectable gene mutants	5 to 15%	2.5 × 10^˗5^ to 2.5 × 10^-4^	Greiner et al. (2017)
	Cas9	Vector driven/RNP	Electroporation	*FKB12, ALS, ARG*	Rapamycin and zeocin	Gene-within-a-gene hybrid construct, composed of a Cas9 gene containing an artificial intron and an inserted sgRNA gene	*Not mention*	3 × 10^-8^	Jiang & Weeks (2017)
	Cpf1 (Cas12a)	RNP	Electroporation	*FKB12, CpFTSY, CpSRP43, PHT7*	Rapamycin and based on coloration and chlorophyll fluorescence	CRISPR/Cpf1-mediated DNA editing efficiencies increased 500-fold with the use of single-stranded oligodeoxynucleotides (ssODNs)	∼10%	*Not mention*	Ferenczi et al. (2017)
	Cas9	RNP	Electroporation	*PLA2*	Hygromycin	Lipid productivity in phospholipase A2 knockout mutants increased by 64.25%, reaching 80.92 g/L/d	*Not mention*	*Not mention*	Shin et al. (2019)
	Cas9	RNP	Electroporation	*ELT1*	Hygromycin	Total lipid accumulated up to 28% of dry biomass, with a 27.2% increase in C18:1 ratio	0.1368	*Not mention*	Nguyen et al. (2020)
	Cas9	RNP	Electroporation	*LCYE, ZEP*	Hygromycin	Mutant with 60% higher zeaxanthin yield (5.24 mg/L) and content (7.28 mg/g)	*Not mention*	*Not mention*	Song et al. (2020)
	Cas9	RNP	Electroporation and glass beads	*IFT81, FAP70, MOT17, CDPK13, CEP131*	Paromomycin	Development of CRISPR-based targeted insertional mutagenesis method (TIM) for *C. reinhardtii*	40% to 95%	*Not mention*	Picariello et al. (2020)
	Cas9	RNP	Cell-penetrating peptide pVEC	*Maa7, FKB12*	Auxotrophic selection using 5-FI (MAA7) and Rapamycin (FKB12)	Delivery of Cas9/sgRNA RNP into *C. reinhardtii* using cell-penetrating peptide pVEC	8.41% to 46.56%	*Not mention*	Kang et al. (2020)
	Cas9	RNP	Electroporation	*PPX1, FTSY, WDTC1*	Paromomycin or oxyfluorfen	Generation of individual strains with precise mutations in multiple target genes	1.3% (*PPX1* and *FTSY*) and 0.8% (*WDTC1*)	*Not mention*	Akella et al. (2021)
	Cas9	RNP	Electroporation	*SPD1*	Paromomycin or hygromycin	Targeted knockout of CrSPD1 induces spermidine auxotroph, which could be used as a selectable marker in biotechnology	10% to 66%	*Not mention*	Freudenberg et al. (2022)
	Cas9	RNP	Glass bead	*NR*	Hygromycin	Generation of mutants with a bacterial phytase gene cassette knocking into the NR gene	0.1481	*Not mention*	Zadabbas Shahabadi et al. (2023)
	Cas9	RNP	Electroporation	*PSR1, CpFTSY*	30% starch	Generation of mutants with impaired extracellular phosphatase synthesis in response to Pi deprivation	0.22	*Not mention*	Battarra et al. (2024)
	Cas9	RNP	Electroporation	*LCYE*	Hygromycin	A 2.3-fold increase in astaxanthin accumulation in the ΔLCYE mutant	0.0417	*Not mention*	Kneip et al. (2024)
*Nannochloropsis* spp.	Cas9	Vector driven	Electroporation	*NR*	Grow normally under NH4Cl but fail to grow under NaNO_3_	Knockout of the NR gene	0.1% to 1%	*Not mention*	Wang et al. (2016)
	Cas9	*In vitro*–synthesized gRNAs/Cas9-expressing line	Electroporation	Insertional mutagenesis of 18 gene of 20 transcription factors	Blasticidin or Hygromycin	Improvement in the total carbon-to-lipid ratio from 20% (wild type) to 40–55% (mutants)	6.25 to 78%	*Not mention*	Ajjawi et al. (2017)
	Cas9	*In vitro*–synthesized gRNAs/reporter-free Cas9-expressing line	Electroporation	*Aco1*	Blasticidin and Hygromycin	Mutants with doubled lipid productivity and ∼50% reduced photosynthetic antenna size	∼50%	*Not mention*	Verruto et al. (2018)
	Cas9 and Cas12a	RNP	Electroporation	*NR*	Zeocin	*Fn*Cas12a generated HDR-based mutants with up to 93% efficiency	34–71% (for Cas9) and 3-93% (for Cas12a)	*Not mention*	Naduthodi et al. (2019)
	Cas9	*In vitro*–synthesized gRNAs/reporter-free Cas9- expressing line	Electroporation	Knock-in of *FAD12* gene at the T1 hotspot	Zeocin	Improved production of polyunsaturated fatty acids (PUFAs)	0.714	*Not mention*	Ryu et al. (2021)
	Cas12a	RNP	Electroporation	*NR*	Grow normally under NH4Cl but fail to grow under NaNO_3_	Generation of markerless genome editing tool to knock out target genes	0.1052	*Not mention*	Naduthodi et al. (2021)
	Cas9	Vector driven	Electroporation	*LER1* and *LER2*	Hygromycin	Double deletion of both *LER1* and *LER2* (from chromosome 9), total ~214 kb	0.285	*Not mention*	Wang et al. (2021)
	dCas9	Vector driven	Electroporation	*g1248*	Zeocin	Growth and photosynthetic parameters (Fv/Fm) of the mutants increased by 23% and 12%, respectively, compared to the wild type under ambient CO_2_ levels	*Not mention*	*Not mention*	Wei et al. (2022)
	Cas9	Vector driven	Bombardment	*LSMT*	Hygromycin	Mutation induced 18–20% reduction in fructose-1,6-bisphosphate aldolases, along with 9.7–13.8% increase in dry weight and enhanced growth	0.1	2 × 10^-8^	Liang et al. (2024)
*Phaeodactylum tricornutum*	Cas9	Vector driven	Bombardment	*CpSRP54*	Zeocin	Generation of stable targeted gene mutations in marine algae	0.31	*Not mention*	Nymark et al. (2016)
	Cas9	Vector-driven and RNP	Bombardment	*PtUMPS, PtAPT, PtAureo1a*	Nourseothricin, 2-FA, adenine, and uracil	A single-step generation of triple knockout strains	65% to 100%	*Not mention*	Serif et al. (2018)
	Cas9	Vector driven	Bacterial conjugation	*Phatr3_J46193*	Phleomycin	Bacterial conjugation-mediated Cas9 delivery to minimize genome exposure to nuclease activity	*Not mention*	2 × 10^-5^	Russo et al. (2018)
	Cas9	Vector driven	Electroporation	*LACS*	Zeocin	Reduced growth rate and altered molecular profiles of PC and TAGs	*Not mention*	*Not mention*	Hao et al. (2022)
	Cas9	Vector driven	Conjugation of plasmids	*CryP*	Zeocin	Increased light-harvesting protein levels in CryP knockout mutants	*Not mention*	*Not mention*	Yang et al. (2022)
	Cas9	Vector driven	Bombardment	*PtTHIC and PtSSSP*	Zeocin	Targeted mutation of the TPP aptamer in the *THIC* gene encoding HMP-P synthase does not affect thiamine biosynthesis in *P. tricornutum*	*Not mention*	*Not mention*	Llavero-Pasquina et al. (2022)
	Cas9	Vector driven	Bombardment	*CpFTSY*	Zeocin	Generation and characterization of CpFTSY mutants	*Not mention*	*Not mention*	Nymark et al. (2023)
	Cas9	Vector driven	Bombardment	*FucT*	Zeocin	Knockout of *PtFucT1* affected *PtGnTI* activity in the complex, converting the N-glycan to a mannose-type N-glycan	0.333	*Not mention*	Xie et al. (2023)
	Cas9	Vector driven	Electroporation	*StLDP*	Zeocin	StLDP functions as an LD scaffold protein in *P. tricornutum*, regulating LD numbers in the stldp mutant and complemented strains	0.8125	*Not mention*	Yoneda et al. (2023)
	Cas9	Vector driven	Bacterial conjugation	*ZEP 2, ZEP3*	No selective pressure	Generation of zep mutants as a platform for diatoxanthin production	*Not mention*	*Not mention*	Graesholt et al. (2024)
*Chlorella* sp.	Cas9	Vector driven	Electroporation	*fad3*	Hygromycin	Mutants have 46% higher lipid accumulation	*Not mention*	*Not mention*	Lin & Ng (2020)
	Cas9	Vector driven and RNP	Bombardment	*NR, APT*	NaNO_2_ and KClO_3_	Generation of auxotrophic strains	*Not mention*	*Not mention*	Kim et al. (2021)
	CRISPRi and CRISPRa	Vector driven	Electroporation	Randomly mediate gene regulation	Hygromycin	Mutants with protein content of 60% to 65% (w/w) of dry cell weight	0.5	*Not mention*	Lin et al. (2022)
	Cas9	Vector driven	Electroporation	*GS*	Hygromycin	Generation of mutants with enhanced biomass, protein, and lutein content	*Not mention*	*Not mention*	Teng & Ng (2023)
	Cas9	Vector driven	Electroporation	*APT*	Hygromycin	Combination of Alcalase treatment with PEG transformation for efficient gene editing in *Chlorella*	*Not mention*	1 × 10^-7^ to 2 × 10^-7^	Kim et al. (2024)
*Porphyridium purpureum*	Cas9	RNP	Bombardment	*CHS1*	No selective pressure	Generation of chlorophyll synthase loss-of-function mutants with increased phycoerythrin levels	*Not mention*	*Not mention*	Jeon et al. (2021)
*Tetraselmis* sp.	Cas9	RNP	Bombardment	*AGP*	No selective pressure	Mutants had 2.7- and 3.1-fold increased lipid content (21.1% and 24.1% of DCW, respectively)	*Not mention*		Chang et al. (2020)
*Euglena gracilis*	Cas9	RNP	Electroporation	*EgGSL2*	No selective pressure	Transgene-free targeted mutagenesis and ssODN-mediated gene knockin	77.7 to 90.1%	*Not mention*	Nomura et al. (2019)
	LbCas12a	RNP	Electroporation	*EgGSL2, EgcrtB*	No selective pressure	High-efficiency genome editing system using direct delivery of LbCas12a RNP complexes	77.2–94.5%	*Not mention*	Nomura et al. (2024)
	Cas9	RNP	Electroporation	Knockout of 16 carotenoid biosynthetic genes present in *E. gracilis*	No selective pressure	Mutants with different carotenoid compositions	*Not mention*	*Not mention*	Tamaki et al. (2023)
*Thalassiosira pseudonana*	Cas9	Vector driven	Bombardment	*Urease*	Nourseothricin	Significant reduction in growth rate and cell size compared to nitrate growth	0.121	8 × 10^-7^	Hopes et al. (2016)
	Cas9	Vector driven	Bombardment	*Sin1*	Nourseothricin	Mutants exhibit reduced biosilica content and morphological aberrations, affecting cell wall strength and stiffness	*Not mention*	*Not mention*	Görlich et al. (2019)
	Cas9 nickase	Vector driven	Bombardment	*TpθCA3*	Nourseothricin	Development of an efficient Cas9 nickase (D10A) system for highly specific indel introduction into target DNA	0.39	3.4 × 10^-7^	Nawaly et al. (2020)

⸶ Mutagenesis efficiency (%) was determined by calculating the proportion of mutants confirmed through genotyping methods, such as Sanger sequencing or polymerase chain reaction (PCR), against the total number of mutants subjected to genotyping.

⸷ Targeted mutagenesis efficiency was defined by the number of mutants confirmed by genotyping in relation to the initial size of the mutant pool.

*Aco1*, acyl-CoA oxidase; *AGP*,ADP-glucose pyrophosphorylase; *ALS*, acetolactate synthase; *ARG*, argininosuccinate lyase; *APT*, adenine phosphoribosyl transferase; CDPK13, calcium-dependent protein kinase 13; *ChlM*, Mg-protoporphyrin IX S-adenosyl methionine O-methyl transferase; *CHS1*, chlorophyll synthase; *CpFTSY*, chloroplast signal recognition particle receptor protein; *CpSRP*, chloroplast signal recognition particle; *CpSRP43*, chloroplast SRP43; CryP, cryptochrome; *ELT1*, esterase/lipase/thioesterase 1; *EgGSL2*, glucan synthase-like 2; *EgcrtB*, phytoene synthase gene; *FAD12*, Δ12-fatty acid desaturases; *FAP 70*, flagella-associated proteins 70; *FucT*, fucosyltransferase; *fad3*, omega-3 fatty acid desaturase; *GS*, glutamate synthase; *IFT*, Intraflagellar transport; *LACS*, Long-chain acyl-CoA synthetases; *LER*, low expression region; *LCYE*, lycopene epsilon cyclase; *LSMT*, rubisco large-subunit methyltransferase; *MAA7*, beta-subunit of tryptophan synthase; *NR*, nitrate reductase; *PEPC1*, phosphoenolpyruvate carboxylase 1; *PLA2*, phospholipase A_2_; *PPX1*, protoporphyrinogen IX oxidase; *PSY*, phytoene synthase-1; *SPD1*, spermidine synthase gene; *Sin1*, silicanin-1; *StLDP*, stramenopile-type LD protein; *TpθCA3*, θ-type carbonic anhydrase; *VGCC*, voltage-gated calcium channel; *ZEP*, zeaxanthin epoxidase; *Phatr3_J46193*, *P. tricornutum* chr9: 533409–537647 locus; g1248: potential methyltransferase responsible for DNA or mRNA methylation.

**Table 2.** Commercially available ready-to-use CRISPR/Cas toolkits and sources

Components	Source/available toolkits	Company
Plasmids	www.addgene.org	Addgene (USA)
	www.snapgene.com	SnapGene Plasmid Database (USA)
	www.genscript.com	GenScript (USA)
	www.idtdna.com	IDT (USA)
Cas9	http://www.toolgen.com/ko	ToolGen, Inc. (Korea)
	https://www.macrogen.com/ko/main	Macrogen, Inc. (Korea)
	https://sg.idtdna.com/page	IDT (USA)
	https://www.neb.com/en	NEB (USA)
	https://www.takara.co.kr/	TaKaRa (Japan)
gRNA	MEGAshortscript^TM^ T7 Kit	Ambion (USA)
	HiScribe T7 RNA Kit	NEB (USA)
	CUGA7 gRNA Synthesis Kit	Nippon Gene (Japan)
	EnGen^®^ sgRNA Synthesis Kit	NEB (USA)
	Guide-it^TM^ sgRNA In Vitro Transcription	TaKaRa (Japan)

**Table 3.** Cas proteins used in plasmid-based CRISPR/Cas system in microalgae

Microalgal strains	Variants of Cas protein	Origin	Codon optimization	PAM	References
*C. reinhardtii*	*Sp*Cas9	*Streptococcus pyogenes*	Yes	NGG	Jiang et al. (2014)
*C. reinhardtii*	*Sa*Cas9 and *Sp*Cas9	*Staphylococcus aureus* and *S. pyogenes*	Yes	NGG	Greiner et al. (2017), Lee et al. (2022)
*C. reinhardtii*	*Sp*Cas9	*S. pyogenes*	Yes	NGG	Jiang & Weeks (2017)
*C. reinhardtii*	Dead *Sp*Cas9(dCas9)	*S. pyogenes*	Maize codon–optimized	NGG	Kao & Ng (2017)
*P. tricornutum*	*Sp*Cas9	*S. pyogenes*	Yes	NGG	Graesholt et al. (2024), Nymark et al. (2023), Nymark et al. (2016), Russo et al. (2018), Serif et al. (2018), Yoneda et al. (2023)
*N. oceanica IMET1*	*Sp*Cas9	*S. pyogenes*	Yes	NGG	Wang et al. (2016)
*N. gaditana*	*Sp*Cas9	*S. pyogenes*	Yes	NGG	Ajjawi et al. (2017), Verruto et al. (2018)
*N. salina*	*Sp*Cas9	*S. pyogenes*	Chlamydomonas-codon optimized	NGG	Ryu et al. (2021)
*N. oceanica*	dCas9	*S. pyogenes*	Yes	NGG	Wei et al. (2022)
*T. pseudonana*	*Sp*Cas9	*S. pyogenes*	Human-codon optimized	NGG	Görlich et al. (2019), Hopes et al. (2016)
*T. pseudonana*	*Sp*Cas9	*S. pyogenes*	Yes	NGG	Nawaly et al. (2020)
*C. vulgaris*	*Sp*Cas9	*S. pyogenes*	Yes	NGG	Kim et al. (2021)
*C. vulgaris*	*Sp*Cas9	*S. pyogenes*	Maize-codon optimized	NGG	Lin & Ng (2020)
*C. sorokiniana*	dCas9	*S. pyogenes*	Maize-codon optimized	NGG	Lin et al. (2022)

**Table 4.** Promoters and Terminals for CRISPR/Cas system in microalgae

	Features	Microalgal strains	Host	References
**Cas9**	pCaMV 35S/tNOS	*C. reinhardtii*	*Cauliflower mosaic virus (CaMV)*	Jiang et al. (2014), Jiang & Weeks (2017), Kao & Ng (2017)
		*C. vulgaris*		Kim et al. (2021), Lin & Ng (2020)
		*C. sorokiniana*		Kim et al. (2024), Lin et al. (2022)
	pHSP70A-RBCS2/RBCS2 3’UTR	*C. reinhardtii*	*C. reinhardtii*	Greiner et al. (2017)
	pPsaD/tPsaD	*C. reinhardtii*	*C. reinhardtii*	Jiang & Weeks (2017)
	pLHCF2/tLHCF1	*P. tricornutum*	*P. tricornutum*	Nymark et al. (2023), Nymark et al. (2016), Russo et al. (2018)
	pVCP /tATUB	*N. oceanica* IMET1	*N. oceanica* IMET1	Wang et al. (2016)
	pRPL24/ tFRD	*N. gaditana*	*N. gaditana*	Ajjawi et al. (2017), Verruto et al. (2018)
	pRibi/tldsp	*N. oceanica*	*N. oceanica*	Liang et al. (2024), Wang et al. (2021), Wei et al. (2022)
	pTpFCP/tNAT	*T. pseudonana*	*T. pseudonana*	Hopes et al. (2016)
	pNR (nitrate reductase promoter)/tNR (nitrate reductase terminator)	*T. pseudonana*	T. pseudonana	Chang et al. (2020), Görlich et al. (2019), Nawaly et al. (2020)
	pFcpB/tFcpA	*P. tricornutum*	*P. tricornutum*	Yang et al. (2022)
**sgRNA**	pU6/T6	*C. reinhardtii*	*Arabidopsis*	Jiang et al. (2014), Jiang & Weeks (2017), Kao & Ng (2017)
		*C. reinhardtii*	*C. reinhardtii*	Greiner et al. (2017), Lee et al. (2022)
		*P. tricornutum*	*P. tricornutum*	Llavero-Pasquina et al. (2022), Nymark et al. (2023), Nymark et al. (2016), Russo et al. (2018), Serif et al. (2018), Yang et al. (2022)
		*T. pseudonana*	*T. pseudonana*	Chang et al. (2020), Görlich et al. (2019), Hopes et al. (2016), Nawaly et al. (2020), Nymark et al. (2016), Yang et al. (2022)
		*C. vulgaris*	*Arabidopsis*	Kim et al. (2021), Lin & Ng (2020)
		*C. sorokiniana*	*Arabidopsis*	Kim et al. (2024), Lin et al. (2022)
	pATPase/tfd	*N. oceanica* IMET1	*N. oceanica* IMET1	Wang et al. (2016)
	pPsaD/tPsaD	*C. reinhardtii*	*C. reinhardtii*	Jiang & Weeks (2017)
	pRibi/tcs	*N. oceanica*	*N. oceanica*	Liang et al. (2024), Wang et al. (2021), Wei et al. (2022)
**Donor DNA**	pβ-tub/trbcS2 (for HygR)	*C. reinhardtii*	*C. reinhardtii*	Nguyen et al. (2020), Shin et al. (2019)
	pVCP/tVCP (for Sh bleR)	*N. oceanica* IMET1	*N. oceanica* IMET1	Naduthodi et al. (2019)
	pβ-tub/tCOP1 (for aphVII)	*C. reinhardtii*	*C. reinhardtii*	Picariello et al. (2020)
	pHSP70A/RBCS2 3’UTR (for bacterial phytase gene)	*C. reinhardtii*	*C. reinhardtii*	Zadabbas Shahabadi et al. (2023)
	pTUB2/tCOP21 (for aphVIII)	*C. reinhardtii*	*C. reinhardtii*	Kneip et al. (2024)
**Selection marker**	pCaMV 35S/tNOS (for HygR)	*C. reinhardtii*	*CaMV*	Jiang et al. (2014), Kao & Ng (2017)
	pFCP/pFCP (for Sh bleR)	*P. tricornutum*	*P. tricornutum*	Nymark et al. (2016)
	pHSP70-RBCS2/RBCS2-3UTR (for aphVIII)	*C. reinhardtii*	*C. reinhardtii*	Kao & Ng (2017)
	pPsaD/tPsaD (for Sh bleR)	*C. reinhardtii*	*C. reinhardtii*	Jiang & Weeks (2017)
	pTCT/ tEIF3 (for blasticidin deaminase)	*N. gaditana*	*N. gaditana*	Ajjawi et al. (2017)
	pEIF3/tFRD (for HygR)	*N. gaditana*	*N. gaditana*	Ajjawi et al. (2017)
	pUEP/tUEP (for Sh bleR)	*N. salina*	*N. salina*	Ryu et al. (2021)

**Promoter and terminator abbreviations:** pCaMV 35S/tNOS, Cauliflower Mosaic Virus 35S promoter/nopaline synthase terminator; pHSP70A-RBCS2/RBCS2 3’UTR, heat shock protein 70A/ribulose-1,5-bisphosphate carboxylase small subunit 2 tandem chimeric promoter /3' untranslated region of ribulose-1,5-bisphosphate carboxylase small subunit 2; pLHCF2/tLHCF1, pVCP/tATUB, violaxanthin/chlorophyll a binding protein promotor/α-tubulin terminator; pRibi/tldsp, ribosomal subunit bidirectional promoter/lipid droplet surface protein terminator; pNR/tNR, nitrate reductase promoter/ nitrate reductase terminator; pATPase/tfd, V-type ATPase promotor/ferredoxin terminator; pRibi/tcs, ribosomal subunit bidirectional promoter/cellulose synthase terminator; pβ-tub/trbcS2, β-tubulin promoter/ribulose-1,5-bisphosphate carboxylase small subunit 2 terminator; pFCP/pFCP, fucoxanthin, chlorophyll a/c-binding protein gene promoter/terminator; pUEP/tUEP, ubiquitin extension protein promoter/terminator. **Gene abbreviations:** HygR, hygromycin B resistance, Sh ble, *Streptoalloteichus hindustanus* bleomycin/zeocin gene resistance; aphVIII, aminoglycoside 3′-phosphotransferase type VIII encoding gene from *Streptomyces rimosus* for paromomycin resistance.
